# AvrRps4 effector family processing and recognition in lettuce

**DOI:** 10.1111/mpp.13233

**Published:** 2022-05-26

**Authors:** Quang‐Minh Nguyen, Arya Bagus Boedi Iswanto, Geon Hui Son, Uyen Thi Vuong, Jihyun Lee, Jin‐Ho Kang, Walter Gassmann, Sang Hee Kim

**Affiliations:** ^1^ Division of Applied Life Science (BK21 Four Program), Plant Molecular Biology and Biotechnology Research Center Gyeongsang National University Jinju Republic of Korea; ^2^ Department of International Agricultural Technology, Institutes of Green‐bio Science and Technology Seoul National University PyeongChang Republic of Korea; ^3^ Department of Agriculture, Forestry and Bioresources and Integrated Major in Global Smart Farm, College of Agriculture and Life Sciences Seoul National University Seoul Republic of Korea; ^4^ Division of Plant Science and Technology, Christopher S. Bond Life Sciences Center and Interdisciplinary Plant Group University of Missouri Columbia Missouri USA; ^5^ Division of Life Science Gyeongsang National University Jinju Republic of Korea

**Keywords:** AvrRps4, effector‐triggered immunity, hypersensitive response, processing, recognition, XopO

## Abstract

During pathogenesis, effector proteins are secreted from the pathogen to the host plant to provide virulence activity for invasion of the host. However, once the host plant recognizes one of the delivered effectors, effector‐triggered immunity activates a robust immune and hypersensitive response (HR). In planta, the effector AvrRps4 is processed into the N‐terminus (AvrRps4^N^) and the C‐terminus (AvrRps4^C^). AvrRps4^C^ is sufficient to trigger HR in turnip and activate AtRRS1/AtRPS4‐mediated immunity in *Arabidopsis*; on the other hand, AvrRps4^N^ induces HR in lettuce. Furthermore, AvrRps4^N^‐mediated HR requires a conserved arginine at position 112 (R112), which is also important for full‐length AvrRps4 (AvrRps4^F^) processing. Here, we show that effector processing and effector recognition in lettuce are uncoupled for the AvrRps4 family. In addition, we compared effector recognition by lettuce of AvrRps4 and its homologues, HopK1 and XopO. Interestingly, unlike for AvrRps4 and HopK1, mutation of the conserved R111 in XopO by itself was insufficient to abolish recognition. The combination of amino acid substitutions arginine 111 to leucine with glutamate 114 to lysine abolished the XopO‐mediated HR, suggesting that AvrRps4 family members have distinct structural requirements for perception by lettuce. Together, our results provide an insight into the processing and recognition of AvrRps4 and its homologues.

## Graphical abstract

In lettuce, effector processing and effector recognition of the AvrRps4 family are uncoupled; different key residues in AvrRps4 and XopO are required for these functions.

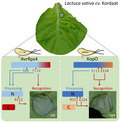

Plant innate immunity relies on a two‐tiered defence response (Chisholm et al., 2006; Dangl et al., 2013; Jones & Dangl, 2006). In the first tier, cell‐surface immune receptors recognize conserved molecular patterns from microbes to launch an induced defence response, called pattern‐triggered immunity (PTI) (Bigeard et al., 2015; Dangl et al., 2013; Jones & Dangl, 2006; Wu & Zhou, 2013). However, pathogens continually compete for domination by secreting a series of effectors to the host plant to suppress PTI (Jones & Dangl, 2006; Lapin & Van den Ackerveken, 2013; Su et al., 2018). As plants and pathogens have coevolved, a second tier of plant immunity has developed based on effector perception by resistance proteins (Andolfo & Ercolano, 2015; Cesari, 2018; Jones & Dangl, 2006). Specifically, resistance proteins directly or indirectly recognize effectors to induce a robust defence, the so‐called effector‐triggered immunity (ETI), that often manifests itself as rapid localized cell death known as the hypersensitive response (HR) (Nguyen et al., 2021; Saur et al., 2021).

An effector named AvrRps4 has been identified in the bacterial pathogen *Pseudomonas syringae* pv. *pisi* (Hinsch & Staskawicz, 1996). AvrRps4 is processed in planta, forming an N‐terminal fragment (AvrRps4^N^) of 133 amino acids and a C‐terminal fragment (AvrRps4^C^) of 88 amino acids (Sohn et al., 2009). The processing of AvrRps4 is dependent on an arginine at position 112 of AvrRps4^N^ (Sohn et al., 2009). AvrRps4 has been described as a bipartite effector (Halane et al., 2018), in which both processed fragments have effector functions. The locus of the resistance gene for AvrRps4, *RPS4*, has been identified, mapped, and characterized (Gassmann et al., 1999; Hinsch & Staskawicz, 1996). This gene encodes a toll/interleukin‐1 receptor nucleotide‐binding leucine‐rich repeat receptor (TNL), which can complement the naturally susceptible phenotype of *Arabidopsis* RLD against bacteria expressing *avrRps4* (Gassmann et al., 1999). *RPS4* transcripts include not only full‐length but also truncated open reading frames generated through alternative splicing activity. The combination of transcripts with full‐length and truncated open reading frames of *RPS4* is required for the recognition of AvrRps4 (Zhang & Gassmann, 2003). AvrRps4 is recognized by the linked gene pairs *RRS1‐RPS4* and/or *RRS1B‐RPS4B* in *Arabidopsis* (Guo et al., 2020; Sarris et al., 2015; Saucet et al., 2015). Conditional overexpression of AvrRps4^C^ in the *Arabidopsis* accession Columbia‐0 (Col‐0) triggers an HR similar to full‐length AvrRps4 (AvrRps4^F^)‐mediated ETI (Li et al., 2014), indicating that the C‐terminus acts as a crucial effector domain of AvrRps4 in *Arabidopsis*. In addition, transient expression of AvrRps4^C^ is sufficient to trigger an HR in turnip (Sohn et al., 2009). While the C‐terminus of AvrRps4 elicits ETI in *Arabidopsis* and turnip, its N‐terminus can be recognized to induce HR in lettuce (*Lactuca sativa* ‘Kordaat’) (Halane et al., 2018; Su et al., 2021). In addition, not only AvrRps4^C^ but also AvrRps4^N^ interacts with EDS1, and both termini are required to trigger immunity in *Arabidopsis* when delivered by bacterial pathogens at natural protein levels (Bhattacharjee et al., 2011; Halane et al., 2018; Heidrich et al., 2011). These findings suggest the function of AvrRps4^N^, which was formerly proposed to only contain a type III secretion system and a chloroplast targeting signal, to be a bona fide effector domain that acts beyond being a signal peptide (Halane et al., 2018, Su et al., 2021).

AvrRps4 has two close homologues, *P. syringae* pv. *tomato* HopK1 and *Xanthomonas campestris* pv. *vesicatoria* XopO (Halane et al., 2018; Li et al., 2014; Sohn et al., 2009; Su et al., 2021), which are together called the AvrRps4 effector family. These three effectors bear a striking similarity in amino acid sequences at the N‐terminal domain, while their C‐terminal domains are unrelated. Like AvrRps4, the N‐terminus of HopK1 (HopK1^N^), but not the C‐terminus of HopK1, triggers an HR in lettuce (Halane et al., 2018), suggesting that the N‐termini of the AvrRps4 family are evolutionarily and functionally identical. Moreover, the processing of AvrRps4 depends on arginine residue 112 (R112) (Sohn et al., 2009), which is conserved among the members of the AvrRps4 family (Su et al., 2021). Interestingly, we found that the R112 residue is important for effector processing and also effector recognition (Su et al., 2021). Indeed, mutation of R112 to leucine (R112L) abolishes AvrRps4‐mediated HR in lettuce. While R112‐mediated AvrRps4^N^ recognition is independent of R112‐mediated AvrRps4^F^ processing, no other AvrRps4 mutation that blocked processing but not recognition, or vice versa, was described by Su et al. (2021). Therefore, due to the dual role of R112, it remained unclear whether AvrRps4^F^ processing is initially required for AvrRps4^N^ recognition. In this study, we found that AvrRps4/XopO processing was not necessary for effector recognition. Furthermore, mutation of the conserved R111 in XopO, if by itself, was insufficient for abolishing XopO recognition in lettuce.

First, we tested whether the N‐terminus of XopO (XopO^N^), which has not been studied previously, could trigger HR in lettuce similarly to AvrRps4^N^ and HopK1^N^. As expected, like AvrRps4^N^ and HopK1^N^, XopO^N^ induced an HR in lettuce cv. Kordaat (Figures 1a and 2a). Furthermore, quantitative ion leakage assays indicated that XopO^N^ elicited an even more robust HR than AvrRps4^N^ and HopK1^N^ at 6 and 11 h (Figure 1b). Then, we compared lettuce responses to AvrRps4, HopK1, XopO, and their mutants to gain an understanding of amino acid features required for effector processing and recognition in lettuce. Through site‐directed mutagenesis, point mutation constructs of the conserved R112 were generated and cloned into the dexamethasone (Dex)‐inducible vector pTA7002. Consistent with the previous study (Su et al., 2021), in our Dex‐inducible system the R112L mutation abolished AvrRps4^N^/AvrRps4^F^‐mediated HR and suppressed electrolyte conductivity in lettuce (Figure S1). Similar to AvrRps4, transient expression of HopK1 constructs showed an identical pattern. HopK1^F (R112L)^ and HopK1^N (R112L)^ successfully suppressed cell death (Figure S2a). Wild‐type HopK1^N^ and HopK1^F^ induced increased electrolyte conductivity, representing stronger HR, compared to their R112 mutations (Figure S2b). Ion leakage from the R112L mutants was statistically slightly higher than that from the negative control. As we expected, the conserved R112 in HopK1 was also important for HopK1^F^ processing (Figure S2c). Surprisingly, a distinct pattern was observed in XopO: as shown in Figure 2a, the R111L mutation in XopO failed to suppress the HR in lettuce. To quantify the amount of cell death, we conducted ion leakage assays. The amount of leakage caused by XopO^N (R111L)^ and XopO^F (R111L)^ was not significantly different compared to that caused by wild‐type XopO^N^ and XopO^F^ (Figure 2b). However, R111L mutation in XopO blocked its processing, like R112 in AvrRps4 and HopK1 effector processing. To further confirm that the XopO^R111L^‐mediated HR is specific, visual cell death assays using mutant sets of N‐terminal or full‐length effectors were performed at the same time. As shown in Figure S4, HR was exclusively observed with XopO^R111L^ mutants. These data indicate that XopO‐mediated HR in lettuce is R111‐independent, and that the HR and effector processing in XopO are uncoupled.

**FIGURE 1:**
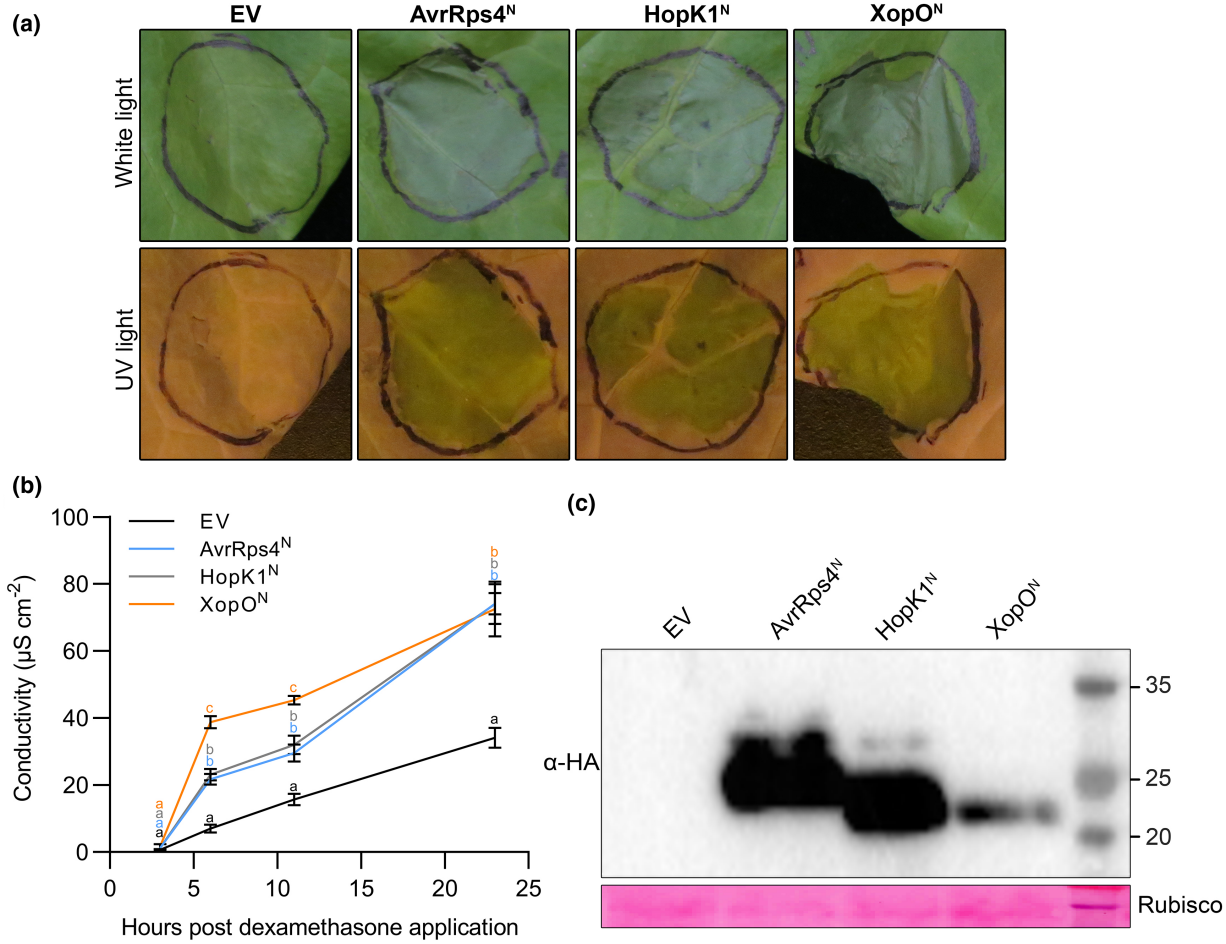
Like AvrRps4^N^ and HopK1^N^, XopO^N^ can trigger a hypersensitive response in *Lactuca sativa* ‘Kordaat’. (a) N‐terminally HA‐tagged proteins and empty vector pTA7002 (EV) were transiently expressed in *L. sativa* ‘Kordaat’ using *Agrobacterium* at an optical density of 0.4. Two days postinfiltration, infiltrated leaves were sprayed with dexamethasone (Dex) solution (50 μM). The photographs were taken under white light and UV light 1 day after Dex treatment. This experiment was repeated twice with identical results. (b) Cell death level was quantified by conductivity as a measure of electrolyte release by cells. Three hours after Dex treatment, lettuce leaf discs were harvested and placed in double‐distilled water containing 0.005% Silwet and 50 μM Dex to initiate measurements. Values represent averages from four replicates and error bars denote *SD*. Two‐way analysis of variance was performed for the statistical tests. Letter codes indicate groups that are significantly different to others according to Tukey's tests (*p* < 0.0001). This experiment was repeated twice with identical results. (c) Protein expression of tested constructs in *L. sativa* ‘Koordat’ was confirmed by western blots. Samples were collected 3 h after Dex treatment. Ponceau S staining confirmed equal loading.

**FIGURE 2:**
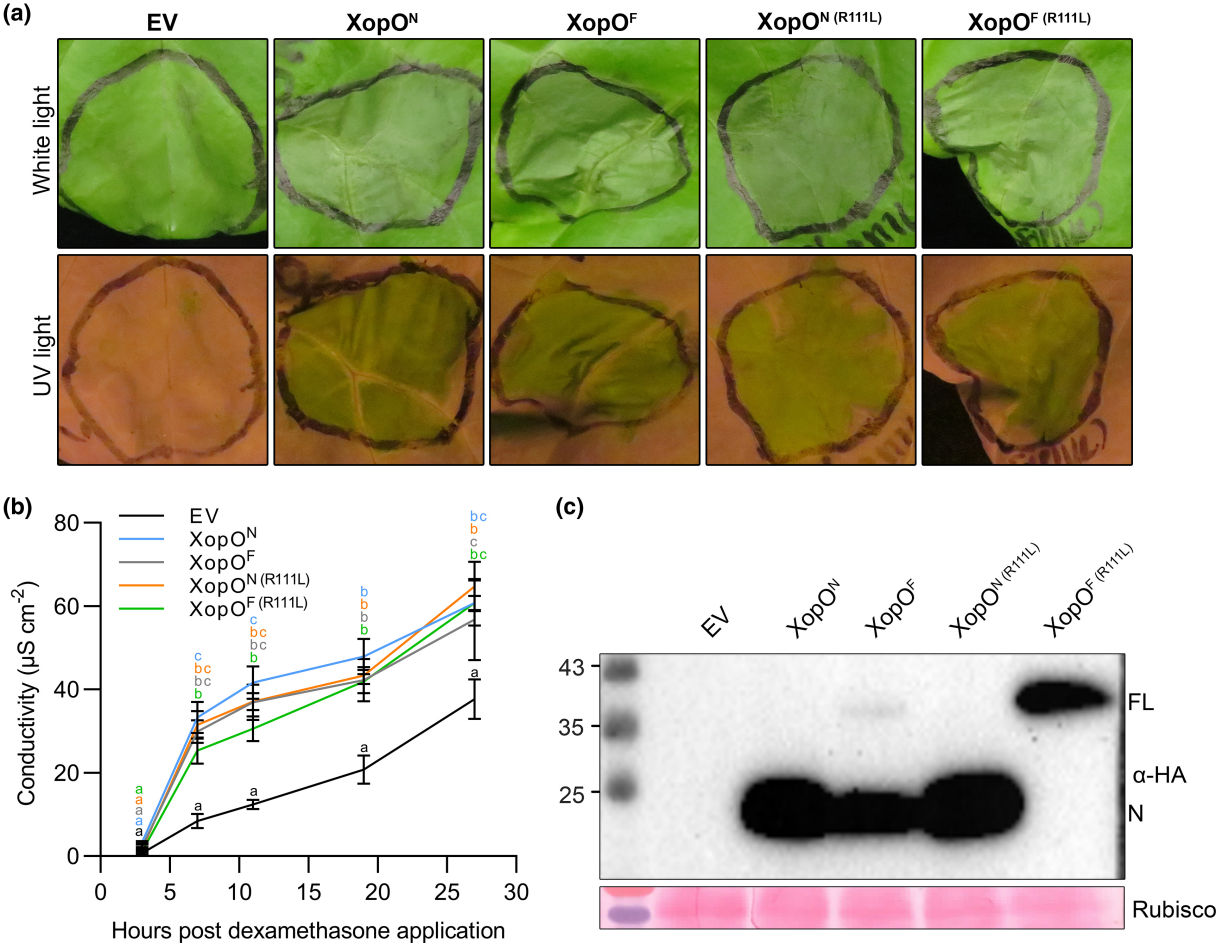
R111L blocks XopO processing but fails to abolish the XopO‐mediated hypersensitive response in *Lactuca sativa* ‘Kordaat’. (a) N‐terminally HA‐tagged proteins and empty vector pTA7002 (EV) were transiently expressed in *L. sativa* ‘Kordaat’, as described in Figure 1. This experiment was repeated twice with identical results. (b) Cell death level was quantified by conductivity as a measure of electrolyte release by cells. Three hours after dexamethasone (Dex) treatment, lettuce leaf discs were harvested and placed in double‐distilled water containing 0.005% Silwet and 50 μM Dex to initiate measurements. Values represent averages from four replicates and error bars denote *SD*. Two‐way analysis of variance was performed for the statistical tests. Letter codes indicate groups that are significantly different to others according to Tukey's tests (*p* < 0.05). This experiment was repeated twice with identical results. (c) Protein expression of tested constructs in *L. sativa* ‘Kordaat’ was confirmed by western blots. Samples were collected 3 h after Dex treatment. Ponceau S staining confirmed equal loading.

Because XopO^(R111L)^ failed to suppress induction of cell death in lettuce, we expected that other or additional residue(s) would play a key role in effector recognition. From the protein alignment (Figure S3), we suspected and highlighted several candidate residues. To test whether another amino acid(s) were crucial for XopO‐ and AvrRps4‐mediated HR in lettuce, we focused on another conserved arginine. Besides R112 in AvrRps4^N^, arginines at position 62 (R62) and position 88 (R88) were conserved among the three effectors (Figure S3). We chose to examine R88 (R87 in XopO) first due to the finding that truncated AvrRps4^N (84–120)^ is sufficient to induce HR (Su et al., 2021). XopO^R87L^ and AvrRps4^R88L^ were generated in a similar way to the construction of the R112 mutants. Using XopO mutants, we found that the R87L single mutant and the R87L/R111L double mutant still induced an HR in lettuce (Figure S5), suggesting that R87 is not required for XopO recognition. In addition, XopO^F (R87L)^ was processed in planta, indicating R87 is not necessary for XopO^F^ processing. The analogous R88L mutation in AvrRps4 also failed to abolish the HR (Figure 3a). However, it surprisingly behaved like R112L to block AvrRps4^F^ processing (Figure 3c). In addition, the level of HR caused by AvrRps4^F (R88L)^ was less than that caused by AvrRps4^F^ (Figure 3b). In the case of AvrRps4^N^ variants, AvrRps4^N (R88L)^ triggered a similar HR level compared to wild‐type AvrRps4^N^ (Figure S6a,b). Equal protein expression levels of AvrRps4^N (R88L)^ and AvrRps4^N^ in protein blot assays confirmed the similarity of HR (Figure S6c). These findings provided strong evidence that effector processing of the AvrRps4 family is not necessary for effector recognition. Effector processing seems to contribute to faster effector recognition because the cell death determinant is located at the N‐terminus, which is processed from the full‐length effector, and because AvrRps4^N^ induces a stronger HR than AvrRps4^F^ (Halane et al., 2018; Su et al., 2021).

Next, we investigated the function of the conserved R62 in AvrRps4. Unlike the conserved R112 and R88 residues, the substitution of R62 to leucine in AvrRps4 did not alter the AvrRps4 processing activity (Figure S7c). In addition, AvrRps4^R62L^ induced an HR in lettuce (Figure S7a,b), indicating that R62 does not function in AvrRps4 recognition. This result is consistent with our previous data that the cell death determinant of AvrRps4 is from residues 84 to 120 (Su et al., 2021). Taken together, our data indicate that effector processing and effector recognition in the AvrRps4 family are uncoupled in lettuce, and different key residues for these are required for these activities.

**FIGURE 3:**
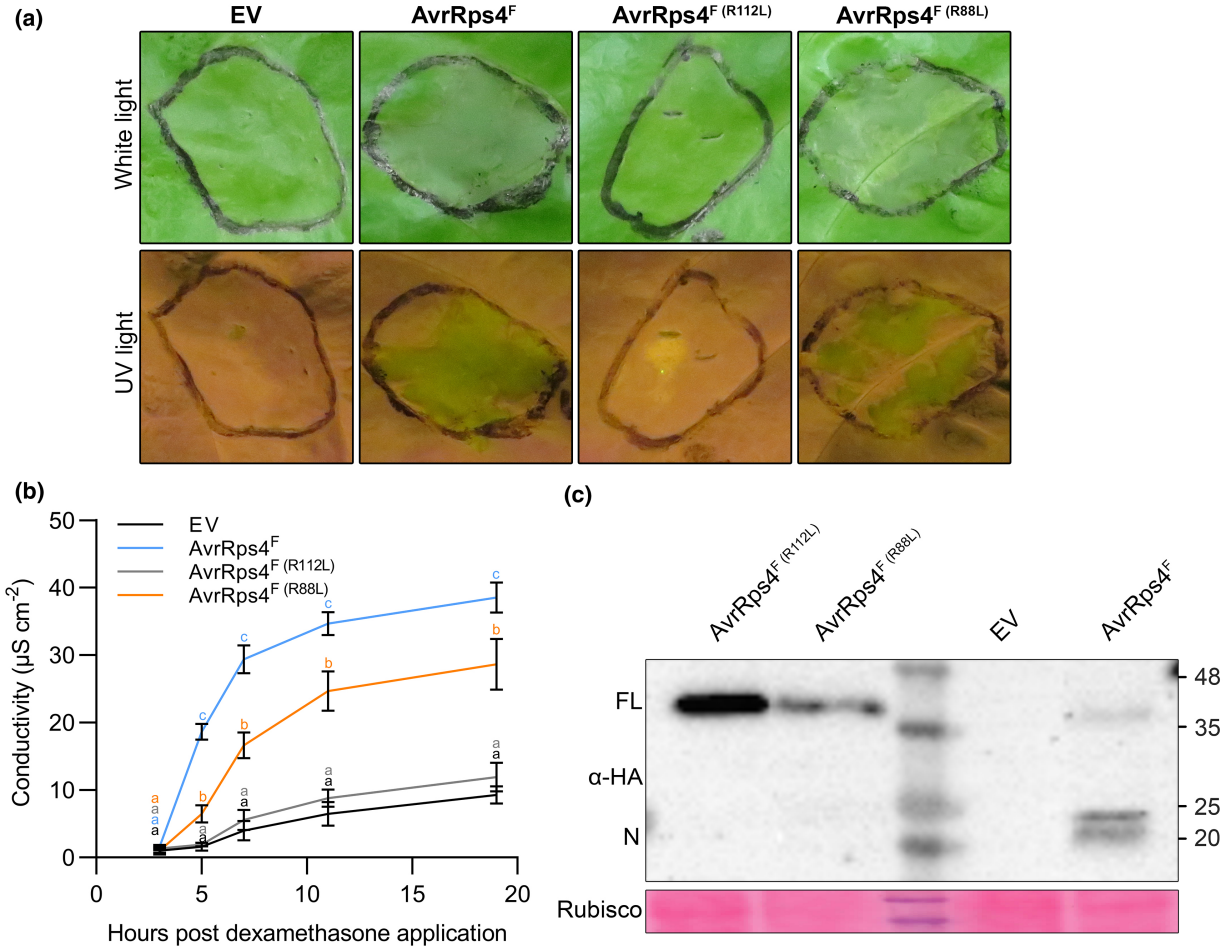
R88L blocks AvrRps4^F^ processing but still triggers a hypersensitive response in *Lactuca sativa* ‘Kordaat’. (a) N‐terminally HA‐tagged proteins and empty vector pTA7002 (EV) were transiently expressed in *L. sativa* ‘Kordaat’, as described in Figure 1. This experiment was repeated twice with identical results. (b) Cell death level was quantified by conductivity as a measure of electrolyte release by cells. Three hours after dexamethasone (Dex) treatment, lettuce leaf discs were harvested and placed in double‐distilled water containing 0.005% Silwet and 50 μM Dex to initiate measurements. Values represent averages from four replicates and error bars denote *SD*. Two‐way analysis of variance was performed for the statistical tests. Letter codes indicate groups that are significantly different to others according to Tukey's tests (*p* < 0.01). This experiment was repeated twice with identical results. (c) Protein expression of tested constructs in *L. sativa* ‘Kordaat’ was confirmed by western blots. Samples were collected 3 h after Dex treatment. Ponceau S staining confirmed equal loading.

Next, we investigated whether residues in addition to the conserved R111 are critical for XopO‐triggered cell death in lettuce. For this, we focused on the AvrRps4 fragment from residues 84 to 120, as this region is necessary and sufficient for the recognition (Su et al., 2021) and is the most conserved region in the alignment of three effector sequences (Figure S3). We aimed to find positions for polymorphic amino acids among the three effector sequences. Because HopK1^R112L^, not XopO^R111L^, behaved similarly to AvrRps4^R112L^ (Figure S4), we hypothesized that the determinant residue(s) for XopO‐triggered cell death should be polymorphic to those that are conserved at the same position between AvrRps4 and HopK1. Moreover, we also hypothesized that the determinant residue(s) in XopO might have a different charge to the conserved residue(s) in AvrRps4 and HopK1. Different charged amino acids can cause different functional characteristics of proteins, which might result in the differential recognition of AvrRps4/HopK1 and XopO by lettuce. Four candidate amino acid residues between residues 84 to 120, were identified: D85_AvrRps4/HopK1_ (K84_XopO_), N107_AvrRps4/HopK1_ (D106_XopO_), Q111_AvrRps4/HopK1_ (E110_XopO_), and K115_AvrRps4/HopK1_ (E114_XopO_) (Figure S3). Among these four, we focused on E110 and E114 in XopO due to their positions being closer to the conserved R111. AvrRps4/HopK1 contain a positive‐charged K115, whereas XopO carries a negative‐charged E114. XopO harbours negative‐charged glutamic acid (E) at position 111, while AvrRps4/HopK1 have a corresponding neutral‐charged glutamine (Q). On the basis of this, we generated a XopO double mutant E110Q/R111L and triple mutant E110Q/R111L/E114K through site‐directed mutagenesis. As shown in Figure S8, the double mutant XopO^E110Q/R111L^ failed to suppress the HR in lettuce. However, the triple mutant XopO^E110Q/R111L/E114K^ successfully abolished the HR, suggesting that E114 is involved in XopO recognition in lettuce. To further test whether E114 is sufficient for the XopO recognition, cell death assays were performed, using the single mutant E114K and double mutants E110Q/E114K and R111L/E114K (Figure 4a,b), with the triple mutant XopO^E110Q/R111L/E114K^ as a positive control of HR abolishment. Like, XopO^R111L^, the single mutant XopO^E114K^ induced an HR in lettuce, indicating that E114 alone is insufficient for XopO recognition. Surprisingly, the double mutant XopO^R111L/E114K^, but not XopO^E110Q/E114K^, suppressed the HR in lettuce (Figure 4a,b). Because XopO^E114K^ mirrors AvrRps4 and XopO^R111L/E114K^ mirrors AvrRps4^R112L^ in cell death induction, we wondered whether the K115E mutation in AvrRps4 could compromise HR suppression by R112L. To determine whether or not this hypothesis was correct, we tested the K115E single and R112L/K115E double mutant of AvrRps4 in cell death assays. The K115E single mutant did not affect AvrRps4‐mediated HR (Figure S9). Like the R112L mutant, the R112L/K115E double mutant failed to induce an HR (Figure S9). Therefore, AvrRps4^R112L/K115E^ does not mirror XopO^R111L^ in HR activation in lettuce, showing that R111 and E114 residues in XopO are specific for XopO recognition in lettuce. Our data indicate that R111 and E114, together, are essential for XopO recognition, suggesting key residues for effector recognition are different between XopO and AvrRps4.

**FIGURE 4:**
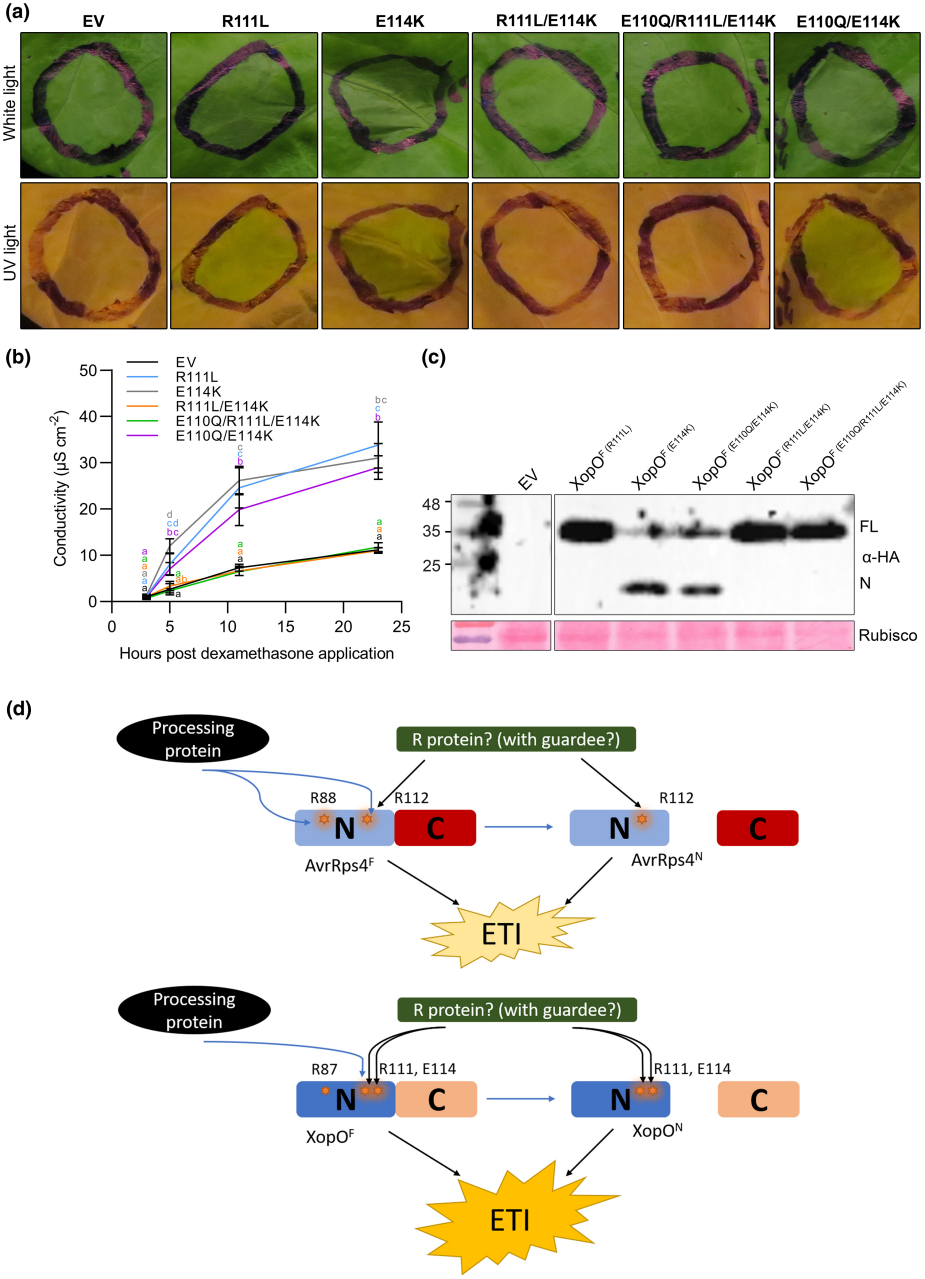
Double mutant R111L, E114K is sufficient to abolish the XopO‐mediated hypersensitive response in *Lactuca sativa* ‘Kordaat’. (a) N‐terminally HA‐tagged proteins and empty vector pTA7002 (EV) were transiently expressed in *L. sativa* ‘Kordaat’, as described in Figure 1. This experiment was repeated twice with identical results. (b) Cell death level was quantified by conductivity as a measure of electrolyte release by cells. Three hours after dexamethasone (Dex) treatment, lettuce leaf discs were harvested and placed in double‐distilled water containing 0.005% Silwet and 50 μM Dex to initiate measurements. Values represent averages from four replicates and error bars denote *SD*. Two‐way analysis of variance was performed for the statistical tests. Letter codes indicate groups that are significantly different to others according to Tukey's tests (*p* < 0.05). This experiment was repeated twice with identical results. (c) Protein expression of tested constructs in *L. sativa* ‘Kordaat’ was confirmed by western blots. Samples were collected 3 h after Dex treatment. Ponceau S staining confirmed equal loading. (d) Proposed models for AvrRps4 and XopO processing and recognition in lettuce. In lettuce, effector processing is not required for effector recognition. The putative resistance protein can recognize both full‐length and N‐terminal effectors. Blue arrows indicate the process of effector processing. Black arrows indicate the process of effector recognition. In AvrRps4, R88 or R112 is important for AvrRps4^F^ processing. However, only R112 is necessary for AvrRps4^N/F^ recognition (top). In XopO, the conserved R111, not the conserved R87, is important for XopO^F^ processing. However, R111 and E114, together, are necessary for XopO^N/F^ recognition. Compared to AvrRps4, XopO is more easily recognized by lettuce due to the two key residues (bottom).

To date, *Arabidopsis* immunity triggered by the C terminus of AvrRps4 has been well studied (Guo et al., 2020; Sarris et al., 2015; Saucet et al., 2015; Sohn et al., 2009). Our previous research raised a new model of AvrRps4‐triggered immunity in lettuce by the N terminus, depending on R112 (Su et al., 2021). Interestingly, HopK1 and XopO, whose conserved R112‐dependent perceptions were not documented before, retain a conserved N‐terminal domain with AvrRps4. This study compared the effector processing and recognition of AvrRps4, HopK1, and XopO. Due to the highly conserved amino acid sequence of the N‐terminal fragment, HopK1 and XopO can induce HR in lettuce, like AvrRps4 (Figures 2, S2, and S3). We presume that a common corresponding resistance protein recognizes the N‐termini of these three effectors in lettuce. AvrRps4/HopK1/XopO‐mediated immunity in lettuce could be a direct recognition or an indirect recognition (with a guardee/decoy) (Figure 4d). The conserved arginine R112 in AvrRps4 plays a critical role in effector processing (Sohn et al., 2009) as well as effector recognition (Su et al., 2021). This binary function of R112 raised the question of whether effector processing is required for effector recognition in lettuce. Effector processing is not required for AvrRps4^C^‐mediated resistance monitored by RRS1/RPS4 in *Arabidopsis* (Sohn et al., 2009). In addition, AvrRps4^F^ mutants impaired in AvrRps4^C^‐triggered immunity do not alter effector processing (Sohn et al., 2012). Moreover, the processing‐impaired mutant AvrRps4^R112L^ still induces AvrRps4^C^ recognition in turnip (Su et al., 2021). In this study, we found another arginine, R88, that is essential for AvrRps4 processing, but not for AvrRps4 recognition, and proved that effector processing was not required for effector recognition: the unprocessed full‐length XopO^R111L^ and AvrRp4^R88L^ were able to induce HR in lettuce (Figures 2 and 3). However, the unprocessed AvrRps4^F (R88L)^ activated a weaker HR than that caused by processed AvrRps4^F^ (Figure 3). It is interesting that processing is not required for the recognition of the C‐terminus in turnip and *Arabidopsis* (Sohn et al., 2009, 2012; Su et al., 2021) nor for the recognition of the N‐terminus in lettuce as shown in this study. Even though effector processing and recognition are uncoupled, we propose that the processing still contributes to a more rapid recognition by producing a structurally smaller and simpler N‐terminus, which is the cell death determinant of the AvrRps4 family in lettuce. Besides the AvrRps4 family, the well‐known effectors AvrRpt2 and AvrPphB also have self‐processing activity (Mudgett & Staskawicz, 1999; Shao et al., 2002). Like AvrRps4, AvrRpt2, a cysteine protease, is inactive outside a host cell. Once injected into host cells, the intrinsic protease activity of AvrRpt2 cleaves off the inhibitory N‐terminus, thus activating AvrRpt2. Following the studies of AvrRpt2 and AvrRps4 processing, we hope to expand research to other unknown/uncharacterized effectors.

So far, we have not found any AvrRps4 family mutant blocked in the recognition but not the processing function. The only mutant we know in AvrRps4 that abolishes the HR in lettuce is AvrRps4^R112L^, which also inhibits its processing. Moreover, the positive charge of R112 is required for R112‐mediated HR (Su et al., 2021). The electric charge of key residues plays an important role in protein function. For instance, negatively charged E175 and E187 in AvrRps4^C^ are critical for AvrRps4‐triggered immunity (Ma et al., 2018; Sohn et al., 2012); electropositive R493 of EDS1 is crucial for TNL‐mediated resistance (Bhandari et al., 2019). Therefore, we suggest that R112 and its positive charge are probably vital for the interaction of AvrRps4 with the resistance protein (or guardee/decoy). Previously we proposed that the conserved R112 may also be functional in AvrRps4 homologues (Su et al., 2021). In this study, we proved that the hypothesis is correct for HopK1 (Figure S2) but not for XopO (Figure 2). Furthermore, we showed that the conserved R111 and nonconserved E114 work together for XopO recognition (Figure 4d). Compared to the positively charged K115 in AvrRps4 and HopK1, we propose that the negative charge of E114 makes a structural difference to XopO, which might lead to a stronger binding to its corresponding immune protein and a more robust HR in lettuce. If R112 is important for an electrostatic interaction of AvrRps4 with another protein, XopO might be able to function without R111 because other unique negatively charged residues are present in the vicinity that may still provide electrostatic interactions with adjacent amino acids in the partner protein. We also suppose that the three effectots (AvrRps4, HopK1, and XopO) were originally recognized identically by lettuce and other unknown plants. Over time, AvrRps4 and HopK1 could have evolutionarily escaped the effector recognition of the unknown plant species by changing the residue 115 to lysine, while XopO did not. However, the single mutation at residue 115 is insufficient for avoiding lettuce perception because R112 is still recognized. In the future, to identify why R111/E114 and only R112 are important for XopO and AvrRps4 recognition, respectively, it is important to perform structural analyses or protein crystallization of AvrRps4^N^ and XopO^N^. This study updates what we know about AvrRps4 homologue effectors regarding their processing and recognition. For further studies, identifying a lettuce bacterial pathogen system for the natural delivery of effectors would be an important tool to characterize structural determinants of immune elicitation by AvrRps4^N^ family members. Beyond the functions of key residues in effector processing and recognition, it would be interesting to discover their effects on the virulence functions of the AvrRps4 family in lettuce and whether effector processing is important for virulence functions. In addition, relative transcript expression of defence marker genes and predicted ETI‐related genes in lettuce in response to AvrRps4 homologues would be of particular interest. More importantly, identification and cloning of the resistance gene (or guardee/decoy) recognizing the AvrRps4 family could help describe the mechanism of AvrRps4/HopK1/XopO‐mediated immunity in lettuce.

## AUTHOR CONTRIBUTIONS

S.H.K. conceived the project. Q.M.N. and S.H.K. designed the experiments. Q.M.N., A.B.B.I., G.H.S., U.T.V., and J.L. performed experiments. Q.M.N. and S.H.K. analysed data and wrote the manuscript. W.G., J.‐H.K., and S.H.K. edited the manuscript. S.H.K. supervised the project.

## Supporting information


**FIGURE S1** R112L abolishes the AvrRps4‐mediated hypersensitive response in *Lactuca sativa* ‘Kordaat’. (a) N‐terminally HA‐tagged proteins and empty vector pTA7002 (EV) were transiently expressed in *L. sativa* ‘Kordaat’ using *Agrobacterium* at an OD of 0.4. Two days postinfiltration, infiltrated leaves were sprayed with dexamethasone (Dex) solution (50 μM). The photographs were taken under white light and UV light 1 day after Dex treatment. (b) Cell death level was quantified by conductivity as a measure of electrolyte release by cells. Three hours after Dex treatment, lettuce leaf discs were harvested and placed in double‐distilled water containing 0.005% Silwet and 50 μM Dex to initiate measurements. Values represent averages from four replicates and error bars denote *SD*. Two‐way analysis of variance was performed for the statistical tests. Letter codes indicate groups that are significantly different to others according to Tukey’s tests (*p* < 0.0001). (c) Protein expression of tested constructs in *L. sativa* ‘Kordaat’ was confirmed by western blots. Samples were collected 3 h after Dex treatment. Ponceau S staining confirmed equal loading.

**FIGURE S2** R112L abolishes the HopK1‐mediated hypersensitive response in *Lactuca sativa* ‘Kordaat’. (a) N‐terminally HA‐tagged proteins and empty vector pTA7002 (EV) were transiently expressed in *L. sativa* ‘Kordaat’, as described in Figure S1. This experiment was repeated twice with identical results. (b) Cell death level was quantified by conductivity as a measure of electrolyte release by cells. Three hours after dexamethasone (Dex) treatment, lettuce leaf discs were harvested and placed in double‐distilled water containing 0.005% Silwet and 50 μM Dex to initiate measurements. Values represent averages from four replicates and error bars denote *SD*. Two‐way analysis of variance was performed for the statistical tests. Letter codes indicate groups that are significantly different to others according to Tukey’s tests (*p* < 0.01). This experiment was repeated twice with identical results. (c) Protein expression of tested constructs in *L. sativa* ‘Koordat’ was confirmed by western blots. Samples were collected 3 h after Dex treatment. Ponceau S staining confirmed equal loading. The asterisk (*) indicates a nonspecific band.

**FIGURE S3** Protein sequence alignment of AvrRps4, HopK1, and XopO using Clustal Omega (https://www.ebi.ac.uk/Tools/msa/clustalo/). An asterisk, a colon and a period illustrate positions that have a fully conserved residue, and conservation between groups of strongly and weakly similar properties, respectively. Numbers refer to the amino acid position of AvrRps4. The black arrow indicates the processing site of effectors in planta. The black box indicates the conserved arginine that is important for effector processing and recognition. Red boxes indicate other conserved arginines in the N‐termini of the three effectors. Blue boxes indicate positions of other residues of interest, which may affect the XopO‐mediated hypersensitive response in *Lactuca sativa* ‘Kordaat’, within the central conserved region from residues 84 to 120 of AvrRps4^N^.

**FIGURE S4** Unlike for AvrRps4 and HopK1, conserved R111L in XopO fails to abolish the hypersensitive response in *Lactuca sativa* ‘Kordaat’. (a) N‐terminally HA‐tagged proteins and empty vector pTA7002 (EV) were transiently expressed in *L. sativa* ‘Kordaat’, as described in Figure S1. (b) Cell death level was quantified by conductivity as a measure of electrolyte release by cells. Three hours after dexamethasone (Dex) treatment, lettuce leaf discs were harvested and placed in double‐distilled water containing 0.005% Silwet and 50 μM Dex to initiate measurements. Values represent averages from four replicates and error bars denote *SD*. Two‐way analysis of variance was performed for the statistical tests. Letter codes indicate groups that are significantly different to others according to Tukey’s tests (*p* < 0.0001).

**FIGURE S5** XopO^F (R87L)^ triggers a similar hypersensitive response level to XopO^F^ in *Lactuca sativa* ‘Kordaat’. (a) N‐terminally HA‐tagged proteins and empty vector pTA7002 (EV) were transiently expressed in *L. sativa* ‘Kordaat’, as described in Figure S1. This experiment was repeated twice with identical results. (b) Cell death level was quantified by conductivity as a measure of electrolyte release by cells. Three hours after dexamethasone (Dex) treatment, lettuce leaf discs were harvested and placed in double‐distilled water containing 0.005% Silwet and 50 μM Dex to initiate measurements. Values represent averages from four replicates and error bars denote *SD*. Two‐way analysis of variance was performed for the statistical tests. Letter codes indicate groups that are significantly different to others according to Tukey’s tests (*p* < 0.05). This experiment was repeated twice with identical results. (c) Protein expression of tested constructs in *Nicotiana benthamiana* was confirmed by western blots. Samples were collected 3 h after Dex treatment. Ponceau S staining confirmed equal loading.

**FIGURE S6** AvrRps4^N (R88L)^ triggers a similar hypersensitive response level to AvrRps4^N^ in *Lactuca sativa* ‘Kordaat’. (a) N‐terminally HA‐tagged proteins and empty vector pTA7002 (EV) were transiently expressed in *L. sativa* ‘Kordaat’, as described in Figure S1. This experiment was repeated twice with identical results. (b) Cell death level was quantified by conductivity as a measure of electrolyte release by cells. Three hours after dexamethasone (Dex) treatment, lettuce leaf discs were harvested and placed in double‐distilled water containing 0.005% Silwet and 50 μM Dex to initiate measurements. Values represent averages from four replicates and error bars denote *SD*. Two‐way analysis of variance was performed for the statistical tests. Letter codes indicate groups that are significantly different to others according to Tukey’s tests (*p* < 0.0001). This experiment was repeated twice with identical results. (c) Protein expression of tested constructs in *L. sativa* ‘Koordat’ was confirmed by western blots. Samples were collected 3 h after Dex treatment. Ponceau S staining confirmed equal loading.

**FIGURE S7** The conserved arginine 62 in AvrRps4 is not required for effector processing or recognition. (a) N‐terminally HA‐tagged proteins and empty vector pTA7002 (EV) were transiently expressed in *Lactuca sativa* ‘Kordaat’, as described in Figure S1. (b) Cell death level was quantified by conductivity as a measure of electrolyte release by cells. Three hours after dexamethasone (Dex) treatment, lettuce leaf discs were harvested and placed in double‐distilled water containing 0.005% Silwet and 50 μM Dex to initiate measurements. Values represent averages from four replicates and error bars denote *SD*. Two‐way analysis of variance was performed for the statistical tests. Letter codes indicate groups that are significantly different to others according to Tukey’s tests (*p* < 0.05). (c) Protein expression of tested constructs in *Nicotiana benthamiana* was confirmed by western blots. Samples were collected 3 h after Dex treatment. Ponceau S staining confirmed equal loading.

**FIGURE S8** E114 is required for XopO‐mediated hypersensitive response in *Lactuca sativa* ‘Kordaat’, while E110 is not. (a) N‐terminally HA‐tagged proteins and empty vector pTA7002 (EV) were expressed in *L. sativa* ‘Kordaat’, as described in Figure S1. (b) Cell death level was quantified by conductivity as a measure of electrolyte release by cells. Three hours after dexamethasone (Dex) treatment, lettuce leaf discs were harvested and placed in double‐distilled water containing 0.005% Silwet and 50 μM Dex to initiate measurements. Values represent averages from four replicates and error bars denote *SD*. Two‐way analysis of variance was performed for the statistical tests. Letter codes indicate groups that are significantly different to others according to Tukey’s tests (*p* < 0.001). (c) Protein expression of tested constructs in *Nicotiana benthamiana* was confirmed by western blots. Samples were collected 3 h after Dex treatment. Ponceau S staining confirmed equal loading.

**FIGURE S9** Like AvrRps4^F (R112L)^, AvrRps4^F (R112L/K115E)^ fails to induce a hypersensitive response in *Lactuca sativa* ‘Kordaat’. N‐terminally HA‐tagged proteins and empty vector pTA7002 (EV) were transiently expressed in *L. sativa* ‘Kordaat’, as described in Figure S1. This experiment was repeated once with identical results.

**FILE S1** Plant materials and growth conditions, plasmid construction, protein sequence alignment, *Agrobacterium*‐mediated infiltration, electrolyte leakage assay, protein extraction, and western blot.

**TABLE S1** Primer sequences used in this study.

## Data Availability

The data that support the findings of this study are available from the corresponding author upon reasonable request.

## References

[mpp13233-bib-0001] Andolfo, G. & Ercolano, M.R. (2015) Plant innate immunity multicomponent model. Frontiers in Plant Science, 6, 987. 26617626 10.3389/fpls.2015.00987 PMC4643146

[mpp13233-bib-0002] Bhandari, D.D. , Lapin, D. , Kracher, B. , von Born, P. , Bautor, J. , Niefind, K. et al. (2019) An EDS1 heterodimer signalling surface enforces timely reprogramming of immunity genes in *Arabidopsis* . Nature Communications, 10, 772. 10.1038/s41467-019-08783-0 PMC6377607 30770836

[mpp13233-bib-0003] Bhattacharjee, S. , Halane, M.K. , Kim, S.H. & Gassmann, W. (2011) Pathogen effectors target *Arabidopsis* EDS1 and alter its interactions with immune regulators. Science, 334, 1405–1408. 22158819 10.1126/science.1211592

[mpp13233-bib-0004] Bigeard, J. , Colcombet, J. & Hirt, H. (2015) Signaling mechanisms in pattern‐triggered immunity (PTI). Molecular Plant, 8, 521–539. 25744358 10.1016/j.molp.2014.12.022

[mpp13233-bib-0005] Cesari, S. (2018) Multiple strategies for pathogen perception by plant immune receptors. New Phytologist, 219, 17–24. 29131341 10.1111/nph.14877

[mpp13233-bib-0006] Chisholm, S.T. , Coaker, G. , Day, B. & Staskawicz, B.J. (2006) Host–microbe interactions: shaping the evolution of the plant immune response. Cell, 124, 803–814. 16497589 10.1016/j.cell.2006.02.008

[mpp13233-bib-0007] Dangl, J.L. , Horvath, D.M. & Staskawicz, B.J. (2013) Pivoting the plant immune system from dissection to deployment. Science, 341, 746–751. 23950531 10.1126/science.1236011 PMC3869199

[mpp13233-bib-0008] Gassmann, W. , Hinsch, M.E. & Staskawicz, B.J. (1999) The *Arabidopsis RPS4* bacterial‐resistance gene is a member of the TIR‐NBS‐LRR family of disease‐resistance genes. The Plant Journal, 20, 265–277. 10571887 10.1046/j.1365-313x.1999.t01-1-00600.x

[mpp13233-bib-0009] Guo, H. , Ahn, H.K. , Sklenar, J. , Huang, J. , Ma, Y. , Ding, P. et al. (2020) Phosphorylation‐regulated activation of the *Arabidopsis* RRS1‐R/RPS4 immune receptor complex reveals two distinct effector recognition mechanisms. Cell Host & Microbe, 27, 769–781 e6. 32234500 10.1016/j.chom.2020.03.008

[mpp13233-bib-0010] Halane, M.K. , Kim, S.H. , Spears, B.J. , Garner, C.M. , Rogan, C.J. , Okafor, E.C. et al. (2018) The bacterial type III‐secreted protein AvrRps4 is a bipartite effector. PLoS Pathogens, 14, e1006984. 29601603 10.1371/journal.ppat.1006984 PMC5895054

[mpp13233-bib-0011] Heidrich, K. , Wirthmueller, L. , Tasset, C. , Pouzet, C. , Deslandes, L. & Parker, J.E. (2011) *Arabidopsis* EDS1 connects pathogen effector recognition to cell compartment‐specific immune responses. Science, 334, 1401–1404. 22158818 10.1126/science.1211641

[mpp13233-bib-0012] Hinsch, M. & Staskawicz, B. (1996) Identification of a new *Arabidopsis* disease resistance locus, *Rps4*, and cloning of the corresponding avirulence gene, *avrRps4*, from *Pseudomonas syringae* pv. *pisi* . Molecular Plant‐Microbe Interactions, 9, 55–61. 8589423 10.1094/mpmi-9-0055

[mpp13233-bib-0013] Jones, J.D. & Dangl, J.L. (2006) The plant immune system. Nature, 444, 323–329. 17108957 10.1038/nature05286

[mpp13233-bib-0014] Lapin, D. & Van den Ackerveken, G. (2013) Susceptibility to plant disease: more than a failure of host immunity. Trends in Plant Science, 18, 546–554. 23790254 10.1016/j.tplants.2013.05.005

[mpp13233-bib-0015] Li, G. , Froehlich, J.E. , Elowsky, C. , Msanne, J. , Ostosh, A.C. , Zhang, C. et al. (2014) Distinct *Pseudomonas* type‐III effectors use a cleavable transit peptide to target chloroplasts. The Plant Journal, 77, 310–321. 24299018 10.1111/tpj.12396

[mpp13233-bib-0016] Ma, Y. , Guo, H. , Hu, L. , Martinez, P.P. , Moschou, P.N. , Cevik, V. et al. (2018) Distinct modes of derepression of an Arabidopsis immune receptor complex by two different bacterial effectors. Proceedings of the National Academy of Sciences of the United States of America, 115, 10218–10227. 30254172 10.1073/pnas.1811858115 PMC6187137

[mpp13233-bib-0017] Mudgett, M.B. & Staskawicz, B.J. (1999) Characterization of the *Pseudomonas syringae* pv. *tomato* AvrRpt2 protein: demonstration of secretion and processing during bacterial pathogenesis. Molecular Microbiology, 32, 927–941. 10361296 10.1046/j.1365-2958.1999.01403.x

[mpp13233-bib-0018] Nguyen, Q.M. , Iswanto, A.B.B. , Son, G.H. & Kim, S.H. (2021) Recent advances in effector‐triggered immunity in plants: new pieces in the puzzle create a different paradigm. International Journal of Molecular Sciences, 22, 4709. 33946790 10.3390/ijms22094709 PMC8124997

[mpp13233-bib-0019] Sarris, P.F. , Duxbury, Z. , Huh, S.U. , Ma, Y. , Segonzac, C. , Sklenar, J. et al. (2015) A plant immune receptor detects pathogen effectors that target WRKY transcription factors. Cell, 161, 1089–1100. 26000484 10.1016/j.cell.2015.04.024

[mpp13233-bib-0020] Saucet, S.B. , Ma, Y. , Sarris, P.F. , Furzer, O.J. , Sohn, K.H. & Jones, J.D. (2015) Two linked pairs of *Arabidopsis* TNL resistance genes independently confer recognition of bacterial effector AvrRps4. Nature Communications, 6, 6338. 10.1038/ncomms7338 25744164

[mpp13233-bib-0021] Saur, I.M.L. , Panstruga, R. & Schulze‐Lefert, P. (2021) NOD‐like receptor‐mediated plant immunity: from structure to cell death. Nature Reviews Immunology, 21, 305–318. 10.1038/s41577-020-00473-z 33293618

[mpp13233-bib-0022] Shao, F. , Merritt, P.M. , Bao, Z. , Innes, R.W. & Dixon, J.E. (2002) A *Yersinia* effector and a *Pseudomonas* avirulence protein define a family of cysteine proteases functioning in bacterial pathogenesis. Cell, 109, 575–588. 12062101 10.1016/s0092-8674(02)00766-3

[mpp13233-bib-0023] Sohn, K.H. , Zhang, Y. & Jones, J.D. (2009) The *Pseudomonas syringae* effector protein, AvrRPS4, requires in planta processing and the KRVY domain to function. The Plant Journal, 57, 1079–1091. 19054367 10.1111/j.1365-313X.2008.03751.x

[mpp13233-bib-0024] Sohn, K.H. , Hughes, R.K. , Piquerez, S.J. , Jones, J.D. & Banfield, M.J. (2012) Distinct regions of the *Pseudomonas syringae* coiled‐coil effector AvrRps4 are required for activation of immunity. Proceedings of the National Academy of Sciences of the United States of America, 109, 16371–16376. 22988101 10.1073/pnas.1212332109 PMC3479578

[mpp13233-bib-0025] Su, J. , Spears, B.J. , Kim, S.H. & Gassmann, W. (2018) Constant vigilance: plant functions guarded by resistance proteins. The Plant Journal, 93, 637–650. 29232015 10.1111/tpj.13798

[mpp13233-bib-0026] Su, J. , Nguyen, Q.M. , Kimble, A. , Pike, S.M. , Kim, S.H. & Gassmann, W. (2021) The conserved arginine required for AvrRps4 processing is also required for recognition of its N‐terminal fragment in lettuce. Molecular Plant‐Microbe Interactions, 34, 270–278. 33147120 10.1094/MPMI-10-20-0285-R

[mpp13233-bib-0027] Wu, Y. & Zhou, J.M. (2013) Receptor‐like kinases in plant innate immunity. Journal of Integrative Plant Biology, 55, 1271–1286. 24308571 10.1111/jipb.12123

[mpp13233-bib-0028] Zhang, X.C. & Gassmann, W. (2003) RPS4‐mediated disease resistance requires the combined presence of RPS4 transcripts with full‐length and truncated open reading frames. The Plant Cell, 15, 2333–2342. 14523247 10.1105/tpc.013474 PMC197299

