# Supplementary material for: AvrRps4 effector family processing and recognition in lettuce
Source: Mol Plant Pathol. 2022 May 26;23(9):1390–8. doi: 10.1111/mpp.13233 (PMC9366065; doi:10.1111/mpp.13233)
Supplement: Supplementary file 1 — FIGURE S1 R112L abolishes the AvrRps4‐mediated hypersensitive response in Lactuca sativa ‘Kordaat’. (a) N‐terminally HA‐tagged proteins and empty vector pTA7002 (EV) were transiently expressed in L. sativa ‘Kordaat’ using Agrobacterium at an OD of 0.4. Two days postinfiltration, infiltrated leaves were sprayed with dexamethasone (Dex) solution (50 μM). The photographs were taken under white light and UV light 1 day after Dex treatment. (b) Cell death level was quantified by conductivity as a measure of electrolyte release by cells. Three hours after Dex treatment, lettuce leaf discs were harvested and placed in double‐distilled water containing 0.005% Silwet and 50 μM Dex to initiate measurements. Values represent averages from four replicates and error bars denote SD. Two‐way analysis of variance was performed for the statistical tests. Letter codes indicate groups that are significantly different to others according to Tukey’s tests (p < 0.0001). (c) Protein expression of tested constructs in L. sativa ‘Kordaat’ was confirmed by western blots. Samples were collected 3 h after Dex treatment. Ponceau S staining confirmed equal loading. [file MPP-23-1390-s010.docx]

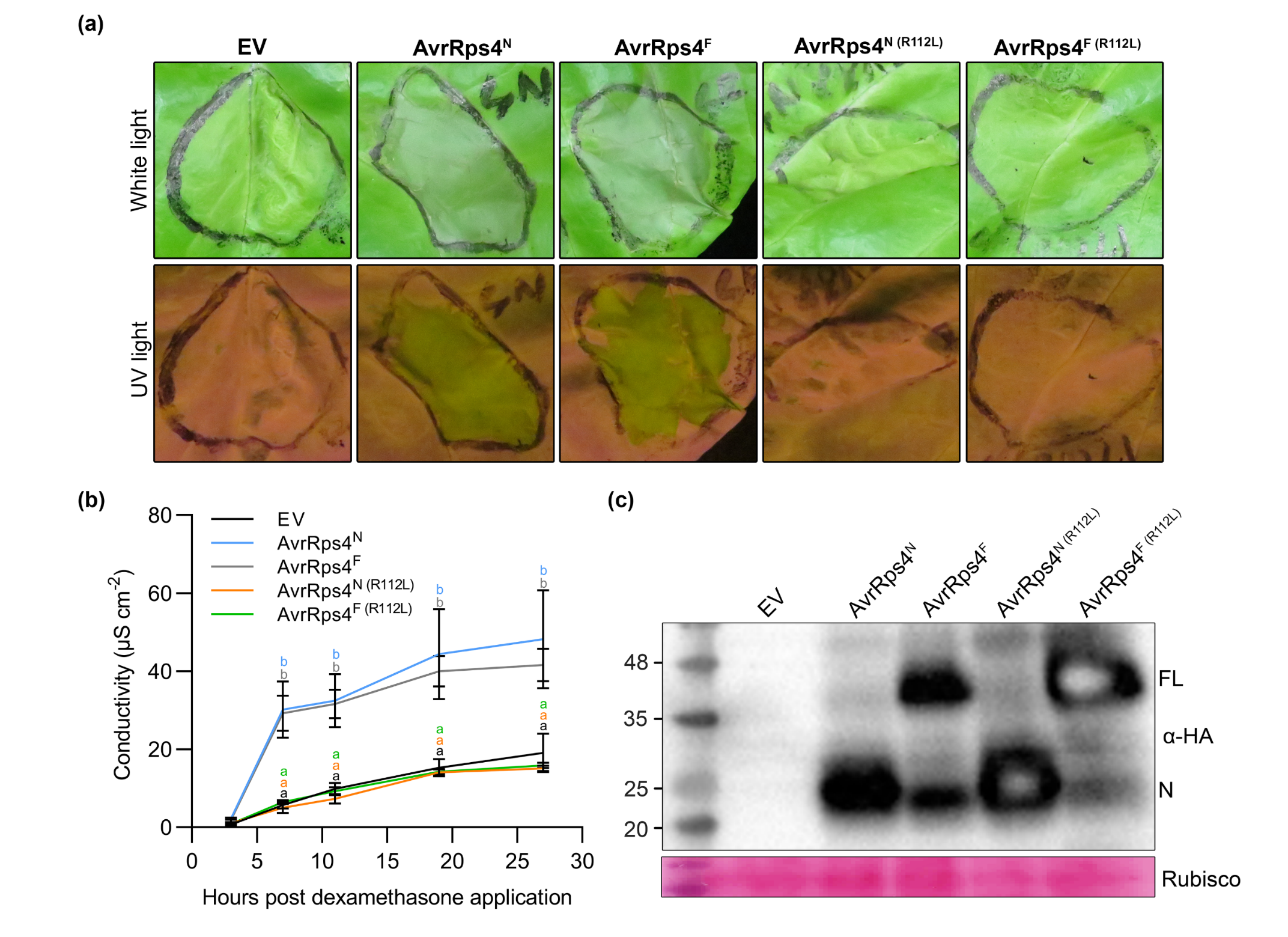


**FIGURE S1** R112L abolishes the AvrRps4-mediated hypersensitive response in *Lactuca sativa* cv. Kordaat.

1. N-terminally HA-tagged proteins and empty vector pTA7002 (EV) were transiently expressed in *L. sativa* cv. Kordaat using agrobacterium at an O.D. of 0.4. Two days post-infiltration, infiltrated leaves were sprayed with Dexamethasone (Dex) solution (50 μM). The photos were taken under white-light and UV-light one day post-Dex treatment.
2. Cell death level was quantified by conductivity as a measure of electrolyte release by cells. Three hours post-Dex treatment, lettuce leaf discs were harvested and placed in ddH2O containing 0.005% Silwet and 50 μM Dex to initiate measurements. Values represent averages from four replicates, and error bars denote SD. Two-way ANOVA analysis was performed for the statistical tests. Letter codes indicate groups that are significantly different to others according to Tukey’s tests (P < 0.0001).
3. Protein expression of tested constructs in *L. sativa* cv. Koordat was confirmed by western blots. Samples were collected three hours post-Dex treatment. Ponceau S staining confirmed equal loading.
