# Supplementary material for: AvrRps4 effector family processing and recognition in lettuce
Source: Mol Plant Pathol. 2022 May 26;23(9):1390–8. doi: 10.1111/mpp.13233 (PMC9366065; doi:10.1111/mpp.13233)
Supplement: Supplementary file 3 — FIGURE S3 Protein sequence alignment of AvrRps4, HopK1, and XopO using Clustal Omega (https://www.ebi.ac.uk/Tools/msa/clustalo/). An asterisk, a colon and a period illustrate positions that have a fully conserved residue, and conservation between groups of strongly and weakly similar properties, respectively. Numbers refer to the amino acid position of AvrRps4. The black arrow indicates the processing site of effectors in planta. The black box indicates the conserved arginine that is important for effector processing and recognition. Red boxes indicate other conserved arginines in the N‐termini of the three effectors. Blue boxes indicate positions of other residues of interest, which may affect the XopO‐mediated hypersensitive response in Lactuca sativa ‘Kordaat’, within the central conserved region from residues 84 to 120 of AvrRps4N. [file MPP-23-1390-s006.docx]

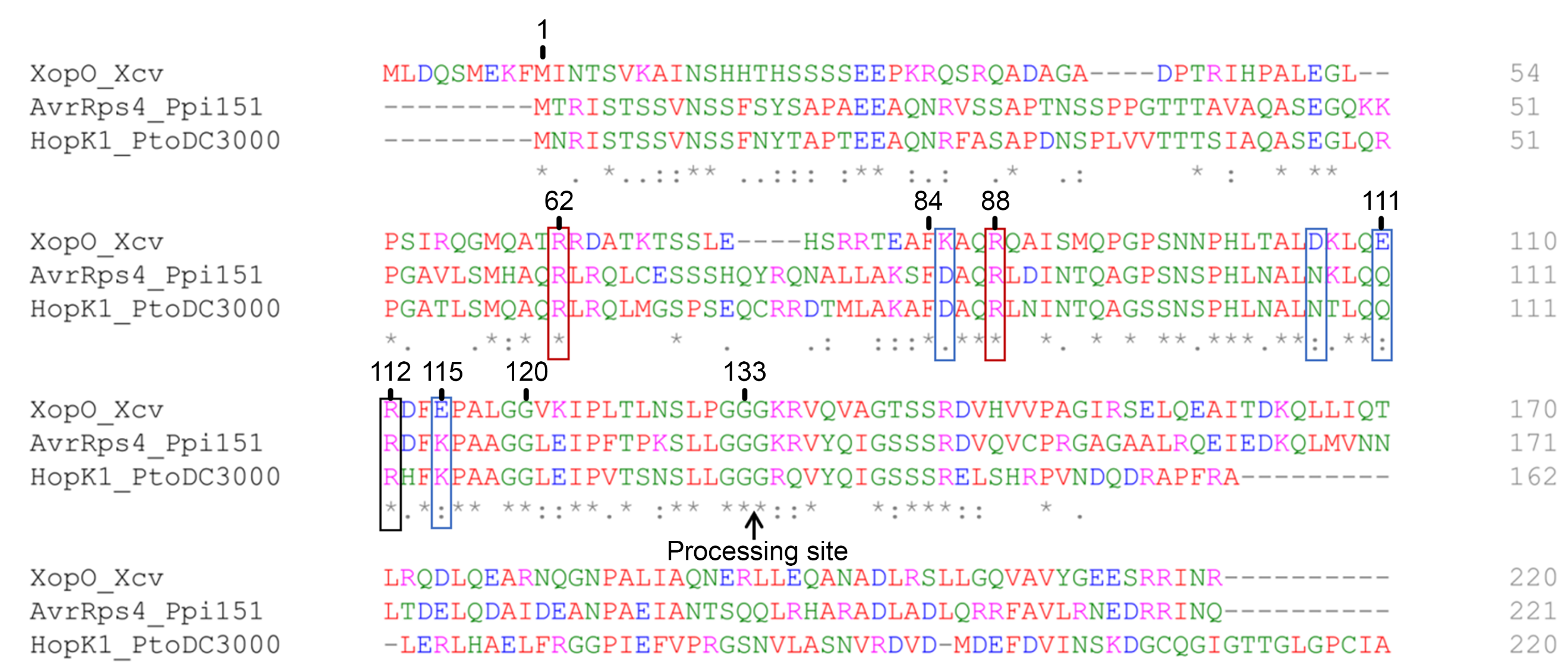


**FIGURE S3** Protein sequence alignment of AvrRps4, HopK1, and XopO using Clustal Omega (https://www.ebi.ac.uk/Tools/msa/clustalo/). An asterisk, a colon and a period illustrate positions that have a fully conserved residue, conservation between groups of strongly and weakly similar properties, respectively. Numbers refer to the amino acid position of AvrRps4. The black arrow indicates the processing site of effectors *in planta*. The black box indicates the conserved arginine, which is important for effector processing and recognition. Red boxes indicate other conserved arginines in the N-termini of the three effectors. Blue boxes indicate positions of other suspect residues, which may involve in XopO-mediated hypersensitive response in *Lactuca sativa* cv. Kordaat, within the central conserved region from residues 84 to 120 of AvrRps4^N^.
