# Supplementary material for: AvrRps4 effector family processing and recognition in lettuce
Source: Mol Plant Pathol. 2022 May 26;23(9):1390–8. doi: 10.1111/mpp.13233 (PMC9366065; doi:10.1111/mpp.13233)
Supplement: Supplementary file 4 — FIGURE S4 Unlike for AvrRps4 and HopK1, conserved R111L in XopO fails to abolish the hypersensitive response in Lactuca sativa ‘Kordaat’. (a) N‐terminally HA‐tagged proteins and empty vector pTA7002 (EV) were transiently expressed in L. sativa ‘Kordaat’, as described in Figure S1. (b) Cell death level was quantified by conductivity as a measure of electrolyte release by cells. Three hours after dexamethasone (Dex) treatment, lettuce leaf discs were harvested and placed in double‐distilled water containing 0.005% Silwet and 50 μM Dex to initiate measurements. Values represent averages from four replicates and error bars denote SD. Two‐way analysis of variance was performed for the statistical tests. Letter codes indicate groups that are significantly different to others according to Tukey’s tests (p < 0.0001). [file MPP-23-1390-s009.docx]

**
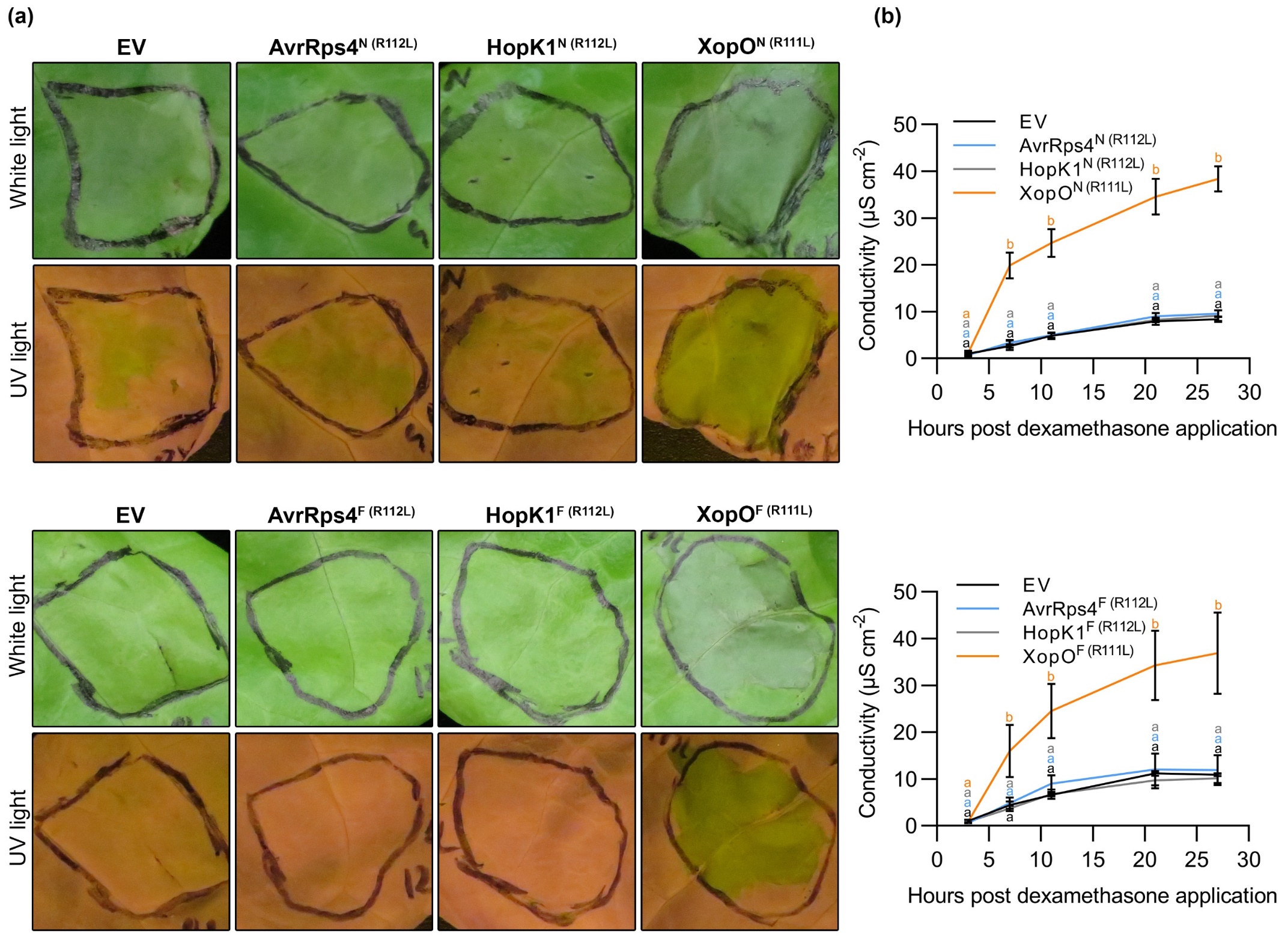
**

**FIGURE S4** Unlike for AvrRps4 and HopK1, conserved R111L in XopO fails to abolish hypersensitive response in *Lactuca sativa* cv. Kordaat.

1. N-terminally HA-tagged proteins and empty vector pTA7002 (EV) were transiently expressed in *L. sativa* cv. Kordaat, as described in Figure S1.
2. Cell death level was quantified by conductivity as a measure of electrolyte release by cells. Three hours post-Dex treatment, lettuce leaf discs were harvested and placed in ddH2O containing 0.005% Silwet and 50 μM Dex to initiate measurements. Values represent averages from four replicates, and error bars denote SD. Two-way ANOVA analysis was performed for the statistical tests. Letter codes indicate groups that are significantly different to others according to Tukey’s tests (P < 0.0001).
