# Supplementary material for: AvrRps4 effector family processing and recognition in lettuce
Source: Mol Plant Pathol. 2022 May 26;23(9):1390–8. doi: 10.1111/mpp.13233 (PMC9366065; doi:10.1111/mpp.13233)
Supplement: Supplementary file 7 — FIGURE S7 The conserved arginine 62 in AvrRps4 is not required for effector processing or recognition. (a) N‐terminally HA‐tagged proteins and empty vector pTA7002 (EV) were transiently expressed in Lactuca sativa ‘Kordaat’, as described in Figure S1. (b) Cell death level was quantified by conductivity as a measure of electrolyte release by cells. Three hours after dexamethasone (Dex) treatment, lettuce leaf discs were harvested and placed in double‐distilled water containing 0.005% Silwet and 50 μM Dex to initiate measurements. Values represent averages from four replicates and error bars denote SD. Two‐way analysis of variance was performed for the statistical tests. Letter codes indicate groups that are significantly different to others according to Tukey’s tests (p < 0.05). (c) Protein expression of tested constructs in Nicotiana benthamiana was confirmed by western blots. Samples were collected 3 h after Dex treatment. Ponceau S staining confirmed equal loading. [file MPP-23-1390-s005.docx]

**
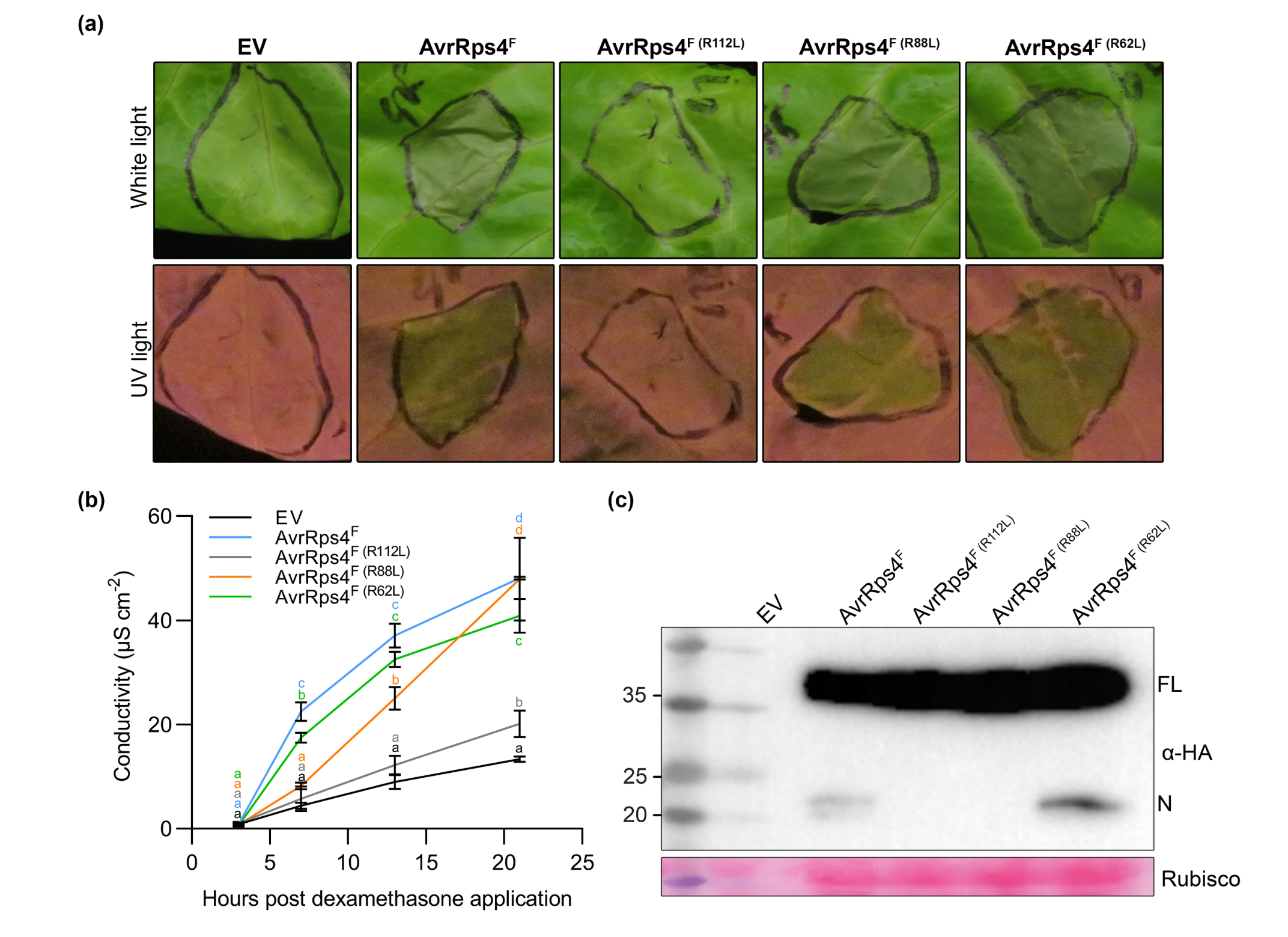
**

**FIGURE S7** The conserved arginine 62 in AvrRps4 is not required for effector processing or recognition.

1. N-terminally HA-tagged proteins and empty vector pTA7002 (EV) were transiently expressed in *L. sativa* cv. Kordaat, as described in Figure S1.
2. Cell death level was quantified by conductivity as a measure of electrolyte release by cells. Three hours post-Dex treatment, lettuce leaf discs were harvested and placed in ddH2O containing 0.005% Silwet and 50 μM Dex to initiate measurements. Values represent averages from four replicates, and error bars denote SD. Two-way ANOVA analysis was performed for the statistical tests. Letter codes indicate groups that are significantly different to others according to Tukey’s tests (P < 0.05).
3. Protein expression of tested constructs in *N. benthamiana* was confirmed by western blots. Samples were collected three hours post-Dex treatment. Ponceau S staining confirmed equal loading.
