# Supplementary material for: AvrRps4 effector family processing and recognition in lettuce
Source: Mol Plant Pathol. 2022 May 26;23(9):1390–8. doi: 10.1111/mpp.13233 (PMC9366065; doi:10.1111/mpp.13233)
Supplement: Supplementary file 9 — FIGURE S9 Like AvrRps4F (R112L), AvrRps4F (R112L/K115E) fails to induce a hypersensitive response in Lactuca sativa ‘Kordaat’. N‐terminally HA‐tagged proteins and empty vector pTA7002 (EV) were transiently expressed in L. sativa ‘Kordaat’, as described in Figure S1. This experiment was repeated once with identical results. [file MPP-23-1390-s007.docx]

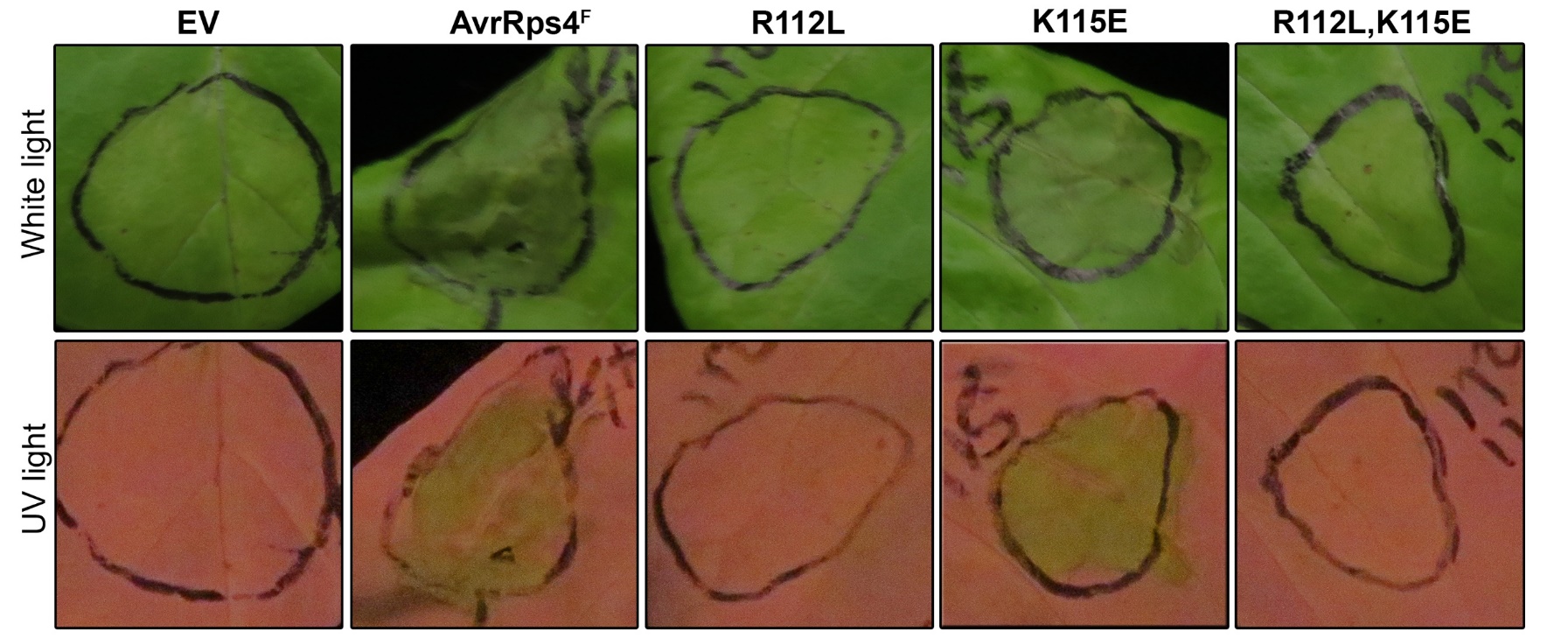


**FIGURE S9** Like AvrRps4^F (R112L)^, AvrRps4^F (R112L/K115E)^ fails to induce a hypersensitive response in *Lactuca sativa* cv. Kordaat.

N-terminally HA-tagged proteins and empty vector pTA7002 (EV) were transiently expressed in *L. sativa* cv. Kordaat, as described in Figure S1. This experiment was repeated once with identical results.
