# Supplementary material for: AvrRps4 effector family processing and recognition in lettuce
Source: Mol Plant Pathol. 2022 May 26;23(9):1390–8. doi: 10.1111/mpp.13233 (PMC9366065; doi:10.1111/mpp.13233)
Supplement: Supplementary file 10 — FILE S1 Plant materials and growth conditions, plasmid construction, protein sequence alignment, Agrobacterium‐mediated infiltration, electrolyte leakage assay, protein extraction, and western blot. [file MPP-23-1390-s002.docx]

**Supporting Information**

**Plant materials and growth conditions.**

Lettuce cultivar Kordaat and *N. benthamiana* were grown in a growth room at 24-26°C and 60% humidity with a 15 h light and 9 h dark cycle for 5-6 weeks.

**Plasmid construction.**

To generate the DEX-inducible gene of interest constructs, a modified multisite Gateway system (Invitrogen) was used, as described previously (Kim et al., 2016). Briefly, the coding sequence of AvrRps4^N/F^, HopK1^N/F^ and XopO^N/F^ were PCR-amplified from a wild-type AvrRps4 and HopK1 plasmid and synthesized XopO sequence templates and were cloned into pDONRII using BP reactions. To make mutations of effectors in pDONRII, PCR-based site-directed mutagenesis were performed, as described previously (Qi and Scholthof, 2008). These entry clones and pDONRI:3×HA were recombined into the pTA7002 destination vector (carrying a DEX-inducible promoter), using LR reactions to generate the N-terminally HA-tagged constructs. All constructs were confirmed by sequencing. Primer sequences used in cloning are listed in Supplementary Table S1.

**Protein sequence alignment.**

Protein sequences for AvrRps4, HopK1 and XopO were aligned using Clustal Omega (<https://www.ebi.ac.uk/Tools/msa/clustalo/>).

**Agrobacterium-mediated infiltration.**

All plasmids in this study were transformed into Agrobacterium tumefaciens C58C1, using electroporation. For HR assays in lettuce, C58C1 carrying corresponding constructs and empty vector were recovered from stock by subculture in Luria-Bertani (LB) liquid medium with appropriate antibiotics. Bacteria were grown at 30 °C with 180 rpm shaking. After overnight culture, cells were collected via centrifugation at 10,000 × rpm at room temperature. Pellets were resuspended in a buffer with 10 mM MgCl_2_ and 200 μM acetosyringone. Suspensions were kept at room temperature for 3 h before adjusting the optical density to 0.4. Then, the suspensions were infiltrated into Lettuce cultivar Kordaat leaves for HR assay and western blot, and *N. benthamiana* leaves for western blot. The infiltrated leaves were sprayed with 50 μM Dexamethasone 48 h post infiltration (hpi). HR phenotypes were first visualized at 6 h after DEX treatment. Photos were taken under UV light and white light 1 day after DEX treatment. For Western blot, tissues were collected 3 h after DEX treatment.

**Electrolyte leakage assay.**

For electrolyte leakage assays, 6 leaf discs were randomly excised and were transferred into 6-well plates containing 5 ml of deionized water with 0.005% Silwet77. The leaf discs were washed twice for 10 min with distilled water. Then, the leaf discs were transferred to 12-well plates containing 5 ml of deionized water with 0.005% Silwet77 and 50 μM Dexamethasone. The conductivity was measured by using Traceable (R) Conductivity/TDS Meter (VWR). Four replicates were measured for each construct. The graphs of electrolyte leakage were generated from software GraphPad Prism 9. The time-point for ion leakage was followed as described in each figure. In each graph, the standard deviation for the error bars and the P values for the statistical tests were conducted by using GraphPad Prism 9. The P values, calculated by the Two-way ANOVA, represent the significant difference of means at each time point.

**Protein extraction and western blot.**

For total protein extraction in tobacco, 6 leaf discs of *N. benthamiana* tissue were ground in 200 µL of 8 M urea buffer, as described previously (Kwon et al., 2009, Halane et al., 2018). For total protein extraction in lettuce, 6 leaf discs of Koordat tissue were ground in 200 µL protein extraction buffer containing 0.1 M Tris-HCl (pH 6.8), 4% (w/v) SDS, 20% (v/v) glycerol, and 0.1 M DTT, as described previously (Su et al., 2021). In both protein extraction methods, plant debris was pelleted at 15,000 rpm for 15 min. The collected supernatant was used for immunoblotting. After adding 5X loading dye to samples, the mixtures were boiled for 10 min. Protein samples were separated on a 13% SDS-polyacrylamide gel and were transferred onto immune-blot PVDF membrane by Trans-blot® Turbo (Bio-Rad, Hercules, Wilmington, DE, USA). Immunodetection was performed with anti-HA-HRP (Roche, Basel, Switzerland) with 1:5000 dilution. Detected proteins were visualized with an ECL Plus chemiluminescent kit (Bio-Rad, Hercules, CA, USA).

HALANE, M. K., KIM, S. H., SPEARS, B. J., GARNER, C. M., ROGAN, C. J., OKAFOR, E. C., SU, J., BHATTACHARJEE, S. & GASSMANN, W. 2018. The bacterial type III-secreted protein AvrRps4 is a bipartite effector. *PLoS Pathog,* 14**,** e1006984.

KIM, S. H., QI, D., ASHFIELD, T., HELM, M. & INNES, R. W. 2016. Using decoys to expand the recognition specificity of a plant disease resistance protein. *Science,* 351**,** 684-7.

KWON, S. I., KIM, S. H., BHATTACHARJEE, S., NOH, J. J. & GASSMANN, W. 2009. SRFR1, a suppressor of effector-triggered immunity, encodes a conserved tetratricopeptide repeat protein with similarity to transcriptional repressors. *Plant J,* 57**,** 109-19.

QI, D. & SCHOLTHOF, K. B. 2008. A one-step PCR-based method for rapid and efficient site-directed fragment deletion, insertion, and substitution mutagenesis. *J Virol Methods,* 149**,** 85-90.

SU, J., NGUYEN, Q. M., KIMBLE, A., PIKE, S. M., KIM, S. H. & GASSMANN, W. 2021. The Conserved Arginine Required for AvrRps4 Processing Is Also Required for Recognition of Its N-Terminal Fragment in Lettuce. *Mol Plant Microbe Interact,* 34**,** 270-278.
