# Supplementary material for: AvrRps4 effector family processing and recognition in lettuce
Source: Mol Plant Pathol. 2022 May 26;23(9):1390–8. doi: 10.1111/mpp.13233 (PMC9366065; doi:10.1111/mpp.13233)
Supplement: Supplementary file 11 — TABLE S1 Primer sequences used in this study. [file MPP-23-1390-s011.docx]

**Table S1** Primer sequences used in the present study

| **Primer Name** | **Sequence (5’-3’)** | **Description** |
| --- | --- | --- |
| AvrRps4-attb4r-F | GGG GAC AAC TTT TCT ATA CAA AGT TGA CAT GAC TCG AAT TTC AAC CAG | For AvrRps4^N/F^ Multisite Gateway cloning |
| AvrRps4^N^-attb2-R | GGG GAC CAC TTT GTA CAA GAA AGC TGG GTA TCC ACC CAA TAG GGA TTT G | For AvrRps4^N^ Multisite Gateway cloning |
| AvrRps4-attb2-R | GGG GAC CAC TTT GTA CAA GAA AGC TGG GTA TTG GTT GAT TCT GCG GTC T | For AvrRps4^F^ Multisite Gateway cloning |
| AvrRps4 (R112L)-F | CAA CTA GAT TTC AAA CCT GCG G | Mutagenesis R112L mutant in AvrRps4^N/F^ |
| AvrRps4 (R112L)-R | GAA ATC TAG TTG TTG GAG CTT G |  |
| AvrRps4 (R88L)-F | CTC AAC TCC TAG ACA TTA ACA CTC | Mutagenesis R88L mutant in AvrRps4^N/F^ |
| AvrRps4 (R88L)-R | CTA GGA GTT GAG CAT CAA AAG |  |
| AvrRps4 (K115E)-F | TTC GAA CCT GCG GCT GG | Mutagenesis K115E mutant in AvrRps4^N/F^ |
| AvrRps4 (K115E)-R | CAG CCG CAG GTT CGA AAT CT |  |
| AvrRps4 (R62L)-F | CAG CTA CTG CGC CAA TTG | Mutagenesis R62L mutant in AvrRps4^N/F^ |
| AvrRps4 (R62L)-R | GCA GTA GCT GTG CAT GCA |  |
| HopK1-attb4r-F | GGG GAC AAC TTT TCT ATA CAA AGT TGA CAT GAA TCG CAT TTC AAC CAG C | For HopK1^N/F^ Multisite Gateway cloning |
| HopK1^N^-attb2-R | GGG GAC CAC TTT GTA CAA GAA AGC TGG GTG ACC GCC CAA TAA GGA GTT G | For HopK1^N^ Multisite Gateway cloning |
| HopK1-attb2-R | GGG GAC CAC TTT GTA CAA GAA AGC TGG GTG GCA GTA GAG CGT GTC GCG AC | For HopK1^F^ Multisite Gateway cloning |
| HopK1 (R112L)-F | CAA CTA CAC TTC AAA CCT GCG G | Mutagenesis R112L mutant in HopK1^N/F^ |
| HopK1 (R112L)-R | GTG TAG TTG TTG GAG CGT G |  |
| attb4r^*^ | GGG GAC AAC TTT TCT ATA CAA AGT TG | For XopO^N/F^ Multisite Gateway cloning |
| XopO^N^-attb2-R | GGG GAC CAC TTT GTA CAA GAA AGC TGG GTA TCC ACC GGG AAG CGA ATT A | For XopO^N^ Multisite Gateway cloning |
| attb2^*^ | GGGG AC CAC TTT GTA CAA GAA AGC TGG GT | For XopO^F^ Multisite Gateway cloning |
| XopO (R111L)-F | GAG CTC GAC TTC GAG CCG | Mutagenesis R111L mutant in XopO^N/F^ |
| XopO (R111L)-R | GTC GAG CTC CTG AAG TTT ATC |  |
| XopO (R87L)-F | CAG CTT CAG GCC ATT TCC A | Mutagenesis R87L mutant in XopO^N/F^ |
| XopO (R87L)-R | CCT GAA GCT GTG CTT TGA AAG |  |
| XopO (E110Q)-F | ACT GCA CTA GAT AAA CTT CAG CAG C | Mutagenesis E110Q mutant in XopO^N/F^ |
| XopO (E110Q)-R | GCT GCT GAA GTT TAT CTA GTG C |  |
| XopO (E114K)-F | TTC AAG CCG GCA CTA GGA | Mutagenesis E114K mutant in XopO^N/F^ |
| XopO (E114K)-R | GTG CCG GCT TGA AGT CG |  |

*: These primers were used to amplify attb4r-XopO-attb2 fragment in the XopO synthesized DNA sequence.
